# Out-of-pocket expenditure by private households for dental services – empirical evidence from Austria

**DOI:** 10.1186/s13561-016-0087-5

**Published:** 2016-03-05

**Authors:** Alice Sanwald, Engelbert Theurl

**Affiliations:** Department of Economics and Statistics, University of Innsbruck, Universitätsstrasse 15, A-6020 Innsbruck, Austria

**Keywords:** Out-of-pocket expenditure, Dental services, Two-part model, Generalized linear model

## Abstract

**Aims:**

Dental services differ from other health services in several dimensions. One important difference is that a substantial share of costs of dental services–especially costs beyond routine dental treatment–is paid directly by the patient out-of-pocket.

**Settings and design:**

This study analyses the socio-economic determinants of out-of-pocket expenditure for dental services (OOPE) in Austria at the household level.

**Methods and material:**

Cross-sectional information on OOPE and household characteristics provided by the Austrian household budget survey 2009/10 was analysed.

**Statistical analysis used:**

A two-part model (Logit/GLM) and one-part GLM was applied.

**Results:**

The probability of OOPE is strongly affected by the life cycle (structure) of the household. It is higher for higher age classes, higher income, and partially higher levels of education. The type of public insurance has an influence on expenditure probability while the existence of private health insurance has no significant effect. In contrast to the highly statistically significant coefficients in the first stage, the covariates of the second stage remain predominantly insignificant. According to the results, the level of expenditure is driven mainly by the level of education and income. The results of the one-part GLM confirm the results of the two-part model.

**Conclusions:**

The results allow new insights into the determinants of OOPE for dental care. The household level turns out to be an adequate basis to study the determinants of OOPE, although caution should be applied before jumping to conclusions for the individual level.

## Background

Dental care services differ to some extent from other medical services, which might influence the mechanisms of service provision and financing. Dental diseases are normally not life threatening and the need for dental services is to some extent predictable and/or preventable. Patients’ ability to learn from experience about provider quality is at least partially possible. However, expenditure smoothing by public and/or private insurance arrangements offer lower opportunities for welfare improvement and higher rates of copayment seem to be optimal. In fact, empirically, out-of-pocket expenditure for dental services (OOPE) is higher compared to other medical services. According to an unweighted OECD average in 2011, OOPE accounted for 53 % of total dental service expenditure, which is roughly three times the level of overall OOPE of healthcare services [1]. In Austria, the situation is similar. OOPE accounts for 50 % of dental service expenditure, leaving 2 % for the general government, 46 % for social health insurance, and 2 % for private health insurance financing in 2011 [2]. This high level of OOPE raises several equity- and efficiency-related questions. From an equity point of view one could ask to which extent OOPE lead to changes in the income distribution on the household level. From an efficiency perspective it is interesting whether OOPE are an adequate tool to reduce moral hazard. However, before drawing any policy conclusions on this equity-efficiency trade off, it seems to be useful to identify the determinants influencing the level of OOPE for dental services. Such an analysis allows deeper insights into the different distributional effects of OOPE beyond the well analysed income dimension (f. e. age structure, household structure, education level, insurance level). This paper focuses on this question at the private household level and analyses cross-sectional information of OOPE and several household characteristics in the latest Austrian household budget survey.

The study benefits from several strands of previous research. It builds on research work on out-of-pocket healthcare expenditure based on micro data in general [3–9], and on the bounded literature on the demand for dental services, in particular on OOPE [10–16]. Finally, the study benefits from research work which focuses on the link between the institutional background of healthcare service consumption and preferred empirical strategies [17–24]. Previous research on the determinants of dental care utilization focuses on different issues. Within the framework of a Becker-type consumer’s choice model Holtman/Olsen [12] study the demand for dental care. They specifically analyse waiting time and travel time as well as the money price as covariates of demand for dental services. Generally, the results confirm the theoretical expectations of the role of the mentioned covariates, but the elasticities are low. Manning/Phelps [13] find a strong effect of insurance coverage on demand for dental care. Groenewegen/Postma [11] stress the role of regional differences in the supply of dental capacities for the utilization of dental services. Their results do not unequivocally support the prediction that the capacity density increases utilization. Nguyen/Häkkinnen [14] investigate the determinants of the utilization of dentists’ services among the Finnish population entitled to subsidized dental care on the basis of age. In particular they focus on the impact of a two-channel financed health care system. They find that the choice between the private and public sector is influenced by the knowledge of the level of dental services provided by each sector. Our study contributes to the empirical research on OOPE for dental services. It adds evidence from the household perspective, completes and adjusts findings available at the individual level, and studies OOPE in a highly particularized healthcare system, which is based on Bismarckian principles and a specific two-tiered institutional architecture. Reliable information on OOPE on a micro basis (individual or household level) is rare in many countries. We use a data basis which in principle offers a high data quality. So as a side effect our paper also evaluates the validity of this data source to study the determinants of health expenditures in general and expenditure for dental services in particular.

The remainder of this paper is organized as follows. The next section briefly describes the institutional setting of consuming dental services in Austria. Subsequently, a brief description of the data, elaboration of the econometric framework, presentation and discussion of the empirical findings, and summary are provided.

### Institutional setting of dental care in Austria

With minor modifications, the general institutional design of demand and supply of outpatient healthcare services in Austria is also relevant for dental services. The social health insurance system represents the first tier of coverage against the risks of illness. Membership in this system is obligatory for wage earners in both the public and private sectors, for self-employed people, and farmers. Individuals with family ties to people with mandatory insurance and without their own coverage obtain free health coverage. Overall, the social health insurance system covers around 99.3 % of the population, excluding only marginal groups. Social health insurance is financed mainly by income-related contributions. Private health insurance and out-of-pocket payments constitute the second tier of the Austrian healthcare system.

Dental services in Austria are offered by (i) private dentists, (ii) public dentists, (iii) dental services offered by the social health insurance system directly (so-called dental laboratories), and (iv) dental ambulances of public and private hospitals. As a workable definition, public dentists are those that have a contract with the social health insurance system. Private and public dentists are self-employed and mainly work in single practices. Patients with social health insurance coverage are free to consult providers of categories (i), (ii), (iii), and, with minor restrictions, also (iv). However, the associated costs of utilization are considerably different.

The consumption of public dental services is based on a benefit-in-kind scheme. Basic dental services (e.g. fillings and teeth extraction) are offered with negligible cost-sharing elements. This is especially true for workers in the private sector (76 % of the population, who are covered by the insurance label GKK). Public workers (8.6 % of the population, who are covered by the insurance label BVA) and employers (8.4 % of the population, who are covered by the insurance label SVA) face a proportional cost-sharing scheme of 20 % for these services, while farmers (4 % of the population, who are covered by the insurance label SVB) have to pay a quarterly lump-sum fee when using dental services, Patients are confronted with substantial amounts of cost sharing (approximately 50 % of the costs) when they undergo specialized treatments, such as endodontic services, crowns and bridges, and prosthodontic and orthodontic services. A closer inspection of the arrangements reveals quite a heterogeneous mix of copayment methods for these dental services (proportional and absolute cost sharing, as well as public subsidies). Cost-sharing designs differ between the different public medical insurance funds, in all of which fixed prosthodontics are cofinanced only by the social health insurance system in exceptional cases.

Similar regulation of service prices and copayments exists for dental services offered by the public health insurance system directly. Dental costs for private dental services are paid out of pocket, and/or by the social health insurance system. The latter reimburses only a portion of a private dentist’s invoice. For basic services, the maximum refundable amount is fixed at 80 % of the amount a public dentist is allowed to charge for the same service. For specialized private treatments, the remuneration schedules of contracted dentists are applied. Since the prices of private dentists for basic and specialized treatments are higher than those for contracted dentists, the financial burden for the utilization of private dentists is substantial. Private health insurance, which in general completes social health insurance coverage in Austria, plays only a very limited role in the coverage of dental expenditure risks. In 2011, 2 % of the total costs for dental services were paid by the private health insurance system [2].

## Methods

To analyse the determinants of OOPE empirically, data from the latest household budget survey 2009/10 conducted by Statistics Austria was used. This periodically repeated survey (at the moment, with a 5-year interval) is used to study the level and structure of private consumption of households within the system of national accounts. The observation unit is the private household without institutionalized households. There is no overlapping in the sample of households, which take part in the different waves of the survey. The total sample offered by Statistics Austria consists of 6534 households with 15,540 members. Owing to unclear household and/or social health insurance status, 747 households were excluded, which results in a final sample size of 5787 households. Information on the consumer behaviour is gathered in two ways: (i) the diary approach and (ii) the recall approach. Households participating in the survey are asked to fill in a diary over 14 days in which they record every single expense. These expenditures are converted into monthly expenditure presented in euros. The dataset results in 52 overlapping weeks of bookkeeping. The recall approach is used for consumer durables and irregular/seasonal expenditures within the last 12 months. In addition, in general, households are asked for expenses greater than 300 € in the last year using the recall method. As far as out-of-pocket expenditures are concerned only information on therapeutic aids in ophthalmology and dentistry is collected by the recall method. Selected socio-economic characteristics of each household are gathered by face-to-face interviews. The household budget survey includes two forms of expenditures for dental care. The first form includes expenditures for dental services in the private and the public sector. These expenditures are mainly for “routine dental services”. The second form of expenditures are expenditures for “specialized treatments”. Specialized treatment mainly includes different forms of dental prostheses (crowns, bridges). In our basic specification we analyse total dental expenditures including routine dental services and specialized treatments. For robustness checks we also estimate the coefficients of the covariates of the two expenditure forms separately. As far as the mode of data gathering is concerned we assume that the information on the expenditures for routine dental services is mainly gathered by the diary approach while information on the expenditures for specialized treatments is collected by the recall method.

For econometric and economic reasons, hurdle models, specifically, two-part models, serve as methodological cornerstones to explain healthcare utilization/expenditure [21]. The first part is a binary model that focuses on the separation between users and nonusers. The second part explains the level/frequency of medical-care use conditional on some use. Statistically, the split in the estimation procedure is motivated by the specific characteristics of healthcare expenditure: (i) skewness, (ii) excess zeros, and (iii) heavy right tails. From an economic perspective, the split in the estimation procedure is motivated by the fact that the two decision stages are characterized by differences in the involved actors and decision covariates. The empirical strategy in the first step normally is based on explicit or reduced versions of the Grossman model of demand for health services [25, 26]. The patient seeking care decides autonomously whether to seek professional diagnostic and curative medical help at all. The modelling of the second step is influenced by principle–agent considerations leading to joint decisions of patients and their service suppliers. In summary, the ideal starting point of two-part models is that the entire episode of medical services is defined as a set of medical services received by a patient in response to particular requests caused by a specific illness (for an extended discussion, see Stoddart and Barer) [24]. The data should portray individual behaviour and should allow separation between the initial spell and additional visits.

The description of the data collection for OOPE in Austria makes clear that the dataset does not perfectly fulfil these preconditions for using a two-part model. Therefore, different econometric approaches were used. First, a two-part model was applied. The first stage of the model predicts the likelihood of any OOPE and was specified as logit leading to the formula:

$$ \Pr ob\left({y}_1>0\right)=\frac{ \exp \left(x\alpha \right)}{1+ \exp \left(x\alpha \right)} $$

The second part predicts the level of spending, conditional on having non-zero OOPE. For the latter part, a generalized linear model (GLM) was used. As an alternative modelling strategy, a one-part GLM and joint estimation of both decision stages was applied [8]. The GLM accommodates skewness and related problems via variance-weighting. In both GLM specifications, the link function and relationship between the mean and variance was determined as suggested by, for example, Manning and Mullahy [27] and Matsaganis et al. [8]. Thereby the mean function is given by:

$$ \mathrm{E}\left(y\Big|x\right)=\mu \left(x\beta \right) $$

If the link function is the log, as it is normally the case in health expenditure applications, then μ is the exponential function. The variance function is normally presented as:

$$ v(x)=\kappa {\left(\mu \left(x\beta \right)\right)}^{\lambda } $$

When λ = 0, the variance is constant, when λ = 1, the variance is proportional to the mean, and when λ = 2 the variance is proportional to the mean squared [8]. In a modified Park test, the squared residuals of a provisional log-transformed ordinary least squares (OLS) model or a provisional GLM model are regressed on the predictions from the same model. The estimated coefficient λ indicates which variance function is appropriate, suggesting either a constant variance model (λ = 0), a proportional to the mean model (λ = 1) or a standard deviation proportional to the mean model (λ = 2). The last two models are sometimes also called ‘Poisson-like’ models or ‘Gamma-like’ models, respectively [17]. As suggested, the goodness of fit of competing model specifications was evaluated by comparing the mean absolute error, mean squared error, and R2 scores [8]. Tests concerning model fit encompass Pregibon’s link test, Ramsey’s regression equation specification error test, a modified Hosmer–Lemeshow test, Cook’s distance, and a goodness of fit test for the combined model.

The dependent variable was defined as monthly OOPE per household and several socio-economic characteristics of the household were used as covariates: household structure or household life cycle, adults’ age structure, adults’ education level, public and private insurance characteristics, sex of the householder, income level, and degree of urbanization. In Table 4 (Appendix), detailed information on the specification of these variables and the percentages of observations with a specific characteristic are given. Table 1 presents the summary statistics of the dependent variable for the explanatory variables and distinguishes between the expenditure means and standard deviation (SD) for the total sample and those households with expenditure higher than zero (1384 households). The average OOPE for the total household size is 35.57 euros (SD = 133.28). The mean for households with non-0 OOPE is 148.74 (SD = 239.72). The data show substantial differences in the OOPE level between households with different characteristics.

**Table 1:** Descriptive statistics according to households’ characteristics and structures

Total households	Dental Care Expenditures				
	Average exp.		Expenditures >0		
	Mean	S.D.	Mean	S.D.	*N*
Household structure					
Single person I	23.75	116.86	141.57	255.19	126
Single person II	24.15	101.76	136.49	208.26	172
Unmarried couple	48.19	236.65	181.64	433.94	91
Married couple	36.12	121.22	165.35	214.82	128
Empty nest	42.77	135.90	149.14	220.51	232
Full nest I	29.74	124.98	129.84	235.45	164
Full nest II	58.80	151.77	167.71	218.01	271
Married couple w/o childs	38.16	116.47	129.61	185.51	106
Single parents	24.52	104.07	122.86	206.25	92
Degree of urbanization					
High urbanization	41.21	158.53	169.37	285.82	502
Average urbanization	36.60	138.14	148.69	246.92	369
Low urbanization	29.65	99.95	128.59	175.06	513
Age structure					
Age <25	7.07	35.41	86.46	94.51	17
Age 25–45	31.13	131.35	134.12	246.17	506
Age 45–65	43.96	144.97	170.07	244.81	588
Age 65–85	32.49	122.11	133.78	219.01	273
Education level					
Primary education	13.15	47.19	74.56	89.96	130
Other education	38.40	140.15	154.93	247.46	1125
Tertiary education	42.79	150.56	169.61	262.05	129
Insurance characteristics (public)					
GKK	30.09	126.79	137.51	242.35	892
BVA	49.75	153.49	174.31	246.88	302
SVA	47.03	127.38	161.43	193.33	141
SVB	46.11	156.38	159.03	259.34	49
Private health insurance (1)	45.56	122.36	179.77	250.98	290
Private health insurance (2)	43.59	145.52	147.91	183.19	207
Total households	35.57	133.28	148.74	239.72	1384
*N* (households)	5787		1384		
Robustness checks					
Specialized treatments	27.41	93.07	122.89	164.66	1261
Routine dental services	8.15	91.62	300.70	472.05	157

Notes: (1) corresponds to one adult of a household that has additional private health insurance. (2) corresponds to both adults of the household having additional private health insurance. This also includes households consisting of one individual (single person I and single person II). Dummy variables for female householders and income are not reported

As a robustness check, OOPE is separated into two components, (i) routine dental services and (ii) specialized treatments (e.g. endodontic services, crowns, and bridges). Overall, 1291 households with positive OOPE are in this expenditure category (the mean OOPE for the total sample is 27.41; the mean OOPE for the sample whose OOPE is more than 0 is 122.88) while only 157 households have positive OOPE for routine dental services (the mean OOPE for the total sample is 8.15; the mean OOPE for the sample whose OOPE is more than 0 is 300.70), see Table 1.

## Results

The econometric results of the two-part-model and the one-part GLM are summarized in Table 2. The probability of having OOPE is influenced strongly by the life cycle of households. In particular, larger observation units, like full nest I (for the specification of the household structure see Appendix Table 4 ), married couples without children, and full nest II, have higher probabilities of spending OOPE. Furthermore, these three household types represent the largest observation units with on average 3.3–4 household members. As only one household member with non-zero OOPE is sufficient to classify the total observation unit as a household that consumes OOPE, the higher probability of the mentioned household types might be explained, at least partially. In addition, there is strong evidence of the relationship between adults’ age and the probability of consuming OOPE. Old age is an important driver of healthcare needs in general, and this is also true for dental healthcare. In the case of the used dataset, the age class of 65–85 years shows the highest probability of OOPE. The type of public insurance influences the probability of OOPE (the reference group is GKK). Households insured by BVA, SVB, and SVA show a higher probability of OOPE, but the results for SVA members remain insignificant. This might reflect the higher proportion of cost sharing in these medical insurance funds. The existence of private health insurance is without any statistically significant effect. Households with a higher level of education and income show a significantly higher probability of having OOPE.

**Table 2:** Econometric results of the two-part model and one-part GLM

	Dental care expenditure					
	Probability (Logit)		Conditional (GLM) ^a^		GLM ^a^	
	Coeff.	Rob. SD	Coeff.	Rob. SD	Coeff.	Rob. SD
Household structure						
Single person II	−0.287*	0.169	−0.005	0.048	−0.154	0.226
Unmarried couple	0.478**	0.194	−0.005	0.052	0.775***	0.266
Married couple	0.125	0.178	−0.068	0.053	−0.137	0.255
Empty nest	0.267	0.186	−0.005	0.049	0.107	0.259
Full nest I	0.390**	0.165	−0.075*	0.043	0.162	0.237
Full nest II	0.690***	0.162	0.008	0.040	0.496**	0.231
Married couple w/o children	0.464**	0.205	−0.081	0.057	−0.040	0.297
Single parents	0.011	0.189	−0.033	0.050	0.012	0.246
Degree of urbanization						
Average urbanization	−0.041	0.095	−0.022	0.024	−0.019	0.136
Low urbanization	−0.135	0.089	−0.022	0.023	−0.287**	0.126
Age structure						
Age 25–45 years	0.997***	0.315	0.062	0.116	1.343***	0.306
Age 45–65 years	1.301***	0.322	0.105	0.117	1.873***	0.321
Age 65–85 years	1.420***	0.341	0.035	0.121	1.861***	0.345
Education level						
Other education	0.272**	0.132	0.089**	0.042	0.694***	0.174
Tertiary education	0.282	0.185	0.090*	0.051	0.832***	0.253
Insurance characteristics (public)						
BVA	0.288***	0.095	0.028	0.025	0.323**	0.139
SVA	0.170	0.129	0.024	0.029	0.304	0.192
SVB	0.531***	0.205	0.066	0.045	0.668**	0.311
Private health insurance (1)	0.179	0.113	0.013	0.026	0.150	0.170
Private health insurance (2)	0.016	0.096	0.007	0.023	0.121	0.134
Other characteristics						
Female householder	0.214**	0.095	0.028	0.025	0.121	0.137
Income (log)	0.235**	0.095	0.129***	0.026	0.466***	0.124
Constant	−4.795***	0.759	0.268	0.218	−2.684***	0.955
Observations (households)	5787		1384		5787	

Notes: ^a^GLM with log-link and gamma distribution. (1) corresponds to one adult of the household with additional private health insurance. (2) corresponds to both adults of the household having additional private health insurance. This also includes households consisting of one individual (single person I and single person II). Reference groups: single person I, high urbanization, age class 18–25 years, primary education, GKK, no additional private health insurance, and male householder. Significance levels: *** *p* < 0.01, ** *p* < 0.05, and * *p* < 0.1

In the second stage, the tested kurtosis verifies a log-link function and the estimated λ clearly suggests a SD proportional to the mean model. The estimated λ of the provisional OLS model with a log-transformed dependent variable has a score of 2.04 and a score of 2.004 in the provisional GLM model. In contrast to the highly statistically significant coefficients in the first stage, the covariates of the second stage remain predominantly insignificant. According to the results, the level of expenditure is driven mainly by the levels of education and income. One explanation is the well-known attitude of both these groups to contact private dentists with higher service fees. Columns 5 and 6 show the results of the one-stage GLM. The tested kurtosis takes the score 3.3, and therefore, justifies a log-link function. The applied Park test shows an estimated λ of 1.84 for the provisional OLS model with a log-transformed dependent variable and an estimated λ of 1.60 for the provisional GLM model. In the evaluation process, the SD proportional to the mean model clearly outperforms the proportional to the mean model, which is used in the subsequent analysis. The considered household types, income, adults’ age, and education level show strong impacts on expenditure level. A negative effect of a lower degree of urbanization is revealed, which might reflect limited access to dental-care facilities. In summary, the findings of the one-stage GLM widely confirm the results the two-part-model.

The econometric results for households consuming specialized treatments are presented in Table 3. The results of the two-part model and the one-part GLM are very similar to the results for total OOPE. The results for routine dental services are widely insignificant and therefore not presented in detail.

**Table 3:** Econometric results of the two-part model and one-part GLM (specialized treatments)

	Expenditures for specialized treatments					
	Probability (Logit)		Conditional (GLM) ^a^		GLM ^a^	
	Coeff.	Rob. S.D.	Coeff.	Rob. S.D.	Coeff.	Rob. S.D.
Household structure						
Single person II	−0.115	0.145	0.121	0.183	−0.010	0.208
Unmarried couple	0.475***	0.174	−0.104	0.174	0.307	0.252
Married couple	0.089	0.158	−0.109	0.180	−0.106	0.243
Empty nest	0.360**	0.159	−0.047	0.175	0.212	0.242
Full nest I	0.353**	0.152	−0.146	0.156	0.084	0.226
Full nest II	0.754***	0.145	0.029	0.142	0.588***	0.218
Married couple w/o childs	0.365**	0.180	−0.421**	0.185	−0.154	0.277
Single parents	0.090	0.165	−0.124	0.160	−0.043	0.227
Degree of urbanization						
Average urbanization	−0.047	0.084	−0.110	0.088	−0.060	0.127
Low urbanization	−0.171**	0.079	−0.147*	0.081	−0.251**	0.117
Age structure						
Age 25–45	1.133***	0.285	0.272	0.342	1.279***	0.292
Age 45–65	1.333***	0.292	0.598*	0.348	1.804***	0.301
Age 65–85	1.488***	0.305	0.522	0.363	1.812***	0.317
Education level						
Other education	0.291**	0.116	0.489***	0.137	0.642***	0.165
Tertiary education	0.220	0.167	0.303*	0.168	0.501**	0.239
Insurance characteristics (public)						
BVA	0.171**	0.085	−0.004	0.080	0.186	0.130
SVA	0.204*	0.113	0.147	0.112	0.338*	0.179
SVB	0.425**	0.182	0.164	0.158	0.436	0.290
Private health insurance (1)	0.220**	0.099	0.024	0.092	0.214	0.159
Private health insurance (2)	0.003	0.084	0.090	0.082	0.024	0.125
Other characteristics						
Female householder	0.093	0.084	0.137	0.099	0.190	0.127
Income (log)	0.202**	0.085	0.475***	0.077	0.610***	0.114
Constant	−4.666***	0.681	0.147	0.657	−3.951***	0.865
Observations (households)	5787		1291		5787	

Notes: ^a^GLM with log-link and gamma distribution. Significance level *** *p* < 0.01, ** *p* < 0.05, * *p* < 0.1

## Discussion

Comparing the results with the findings of previous research [10–16] is only partially useful. This study analyses OOPE. The vast majority of previous studies analyse utilization, measured by visits or total dental expenditure. In addition, the focus of this study is on the household while previous research is based on individual data. Finally, the focus of this study is on socio-economic household characteristics as explanatory variables, and controlling for dental health status and supply-related characteristics in detail is impossible. However, the ‘degree of urbanization’ was used as a proxy for access to dental service. Therefore, this study abstains from drawing any conclusions related to supply side from the results (see Nguyen and Häkkinen [14]). Previous evidence sometimes points to a U-shaped relationship between age and dental utilization/expenditure. The shown effect of age on OOPE is higher in older age classes, which does not contradict a U-shaped relationship. The reference group consists of adults who are on average below 25 years of age. Children are included only in the household structure. Compared to Choi [10], the study presents new and dissenting findings on the role of public and private insurance characteristics on OOPE. The type of public insurance influences OOPE. Copayment mechanisms for routine dental services and, in particular, for special treatment differ between the public medical insurance funds. This is an essential feature of the Austrian healthcare system in general, although movements to harmonize the remuneration system of public dentists and the copayment schemes for specialized treatment are progressing. Of course, differences in the OOPE levels of members of the different public medical insurance funds might also be caused by unobserved heterogeneity between the members of the different insurance groups. Additional private health insurance is without any effect on OOPE. This could be interpreted as an indication that there is a sufficient public level of coverage against dental expenditure risks.

We are cautious in drawing straight conclusions for dental health policy from our empirical findings. In fact we will briefly discuss the question whether data from household budget surveys are an adequate data basis to study the determinants of health care expenditures in general and expenditures for dental care in particular. Reliable data on OOPE are rare, their acquisition is very costly. Health related data sources (ATHIS, SHARE) normally use contacts with the health care system as an indicator for utilization. So the periodically repeated household budget surveys are an alternative information source. Overall the design of household budget surveys offers a high data quality. This is ensured via the level of instructions for the participants and the combination of the diary system and the recall approach. Potential underreporting of the levels of OOPE is reduced by the use of a disaggregated approach that asks for several OOPE categories. On the other hand household budget surveys also have clear limitations. They only include rudimental information on socio-economic characteristics of the household and its members which are important for explaining the utilization of dental services. The same is true for covariates which picture the supply side of dental services (f. e. density of private and public dentists). Additionally, the two modes of data gathering have consequences for the reliability of the empirical methods. In principle, the combination of the diary system for routine expenditures and the recall system for specialized treatment seems to be an adequate strategy of data collection. But we should be aware of the fact that the length of the observation period (2 weeks in the diary system, 1 year in the recall system) has direct consequences for the empirical results in the two steps of the two part model. Finally the information from the household budget survey is period based and does not allow a separation in different steps of the utilization process. Such a separation is necessary to substantiate the two steps of the two part model from an economic point of view.

## Conclusions

This paper analyses the socio-economic determinants of OOPE of private households in Austria using data from the household budget survey 2009/10. The main conclusions are as follows. The characteristics of the data (household-level data, period-based data, and short observation period) pose several challenges for the choice of empirical estimation procedure. So we decided to compare different econometric procedures (two part model, GLM). Oure estimation reveals highly significant results for several household characteristics (household life cycle, adults’ age, adults’ education, and income) in explaining the probability of OOPE (first stage). However, in the second stage (expenditure level), only income and education have significant coefficients. The one-part GLM estimation confirms the results of the two-part model. The existence of private health insurance has no influence on the expenditure probability/level while the type of public insurance influences the expenditure probability. The household structure seems to have a strong effect on the expenditures for specialized treatments. The majority of the covariates used to explain expenditures for routine dental services are widely insignificant. The household turns out to be a promising basis to study the determinants of dental expenditure and supplements the previous research, which focuses on the individual level.

## Appendix

**Table 4:** Overview of variable specification and corresponding share of observations

Variables	Percentage of observations	S.D.	Definition
Household structure			
Single person 1	12.98	0.44	Household consists of 1 adult, single.
Single person II	16.80	0.49	Household consists of 1 adult, either married, divorced or widowed.
Unmarried couple	5.93	0.31	Household consists of 2 adults, unmarried.
Married couple	10.13	0.40	Household consists of 2 adults, married, members are below 60 years.
Empty nest	13.98	0.46	Household consists of 2 adults, married, members are above 60 years.
Full nest 1	12.37	0.43	Household consists of 2 adults, members are below 40 years, at least one child.
Full nest II	13.36	0.45	Household consists of 2 adults, members are above 40 years, at least one child.
Married couple w/o childs	6.22	0.32	Household consists of more than 3 adults, married, no children.
Single parents	7.97	0.36	Household consists of one adult, at least one child,
Degree of urbanization			
High urbanization	35.65	0.63	Areas with a population of at least 50,000 and more than 500 inhabitants per square kilometer.
Average urbanization	25.90	0.58	Areas with a population of at least 50,000 and 100–500 inhabitants per square kilometer.
Low urbanization	38.45	0.54	All other areas.
Age structure			
Age <25	3.59	0.24	Average age of both adults. Refers to householder, if household consists of one adult.
Age 25–45	37.67	0.64	Average age of both adults. Refers to householder, if household consists of one adult.
Age 45–55	39.31	0.64	Average age of both adults. Refers to householder, if household consists of one adult.
Age 65–85	19.42	0.52	Average age of both adults. Refers to householder, if household consists of one adult.
Education level			
Primary education	12.74	0.44	Both adults have a primary education level. This also includes households consisting of one adult.
Other education	78.43	0.54	Both adults have a mixed or secondary education level. This also includes households consisting of one adult.
Tertiary education	8.83	0.37	Both adults have a secondary education level. This also includes households cons/sting of one adult.
Insurance characteristics (public)			
GKK	70.43	0.60	Workers in the private sector. Refers to householder’s insurance type:
BVA	18.28	0.51	Public servants. Refers to householder’s insurance type.
SVA	8.36	0.36	Employers. Refers to householders insurance type.
SVB	2.92	0.22	Farmers. Refers to householder’s insurance type
Additional private health insurance (1)	11.61	0.42	One adult of the household has an additional health insurance.
Additional private health insurance (2)	20.67	0.53	All adults have an additional health insurance. This includes households consisting of one adult.
Other characteristics			
Female householder	32.73	0.62	Householder is female.
Income	2986.40	2025.56	Monthly household Income in Euros.
